# How to Help the Poor

**Published:** 1857-12

**Authors:** 


One of the greatest demoralizations of every day occurrence is a *gift* to poverty. It degrades the receiver on the instant; and every time it is repeated, that degradation is made the more certain and the more complete. But to teach a poor man how to help himself, how to get along, that is a truer charity than all, that is an act of brotherhood! The wide world over, the people who are helped the most, are of the least account. "Help yourself!" is the universal instinct of mere animal life; and man, the nobler animal, is no exception to that world-wide law. If an intelligence office were established in New York, where *tidy*, *educated* girls could be found, who would be willing to work for a dollar a week, there are thousands of humane housekeepers who would cheerfully employ them, and, in addition, aid them in qualifying themselves for a better discharge of their duties.

Another humanity, and a civic economy too, would be for the city authorities, co-operating with private benevolence, to raise a large fund—a generous hundred thousand dollars at once—for the purchase of railroad tickets at a low rate, which should carry the bearer at least six hundred miles into the interior of the country, to be given to indigent persons, so as to convey them to places where provisions are abundant, where help is needed, and so far away, too, that they would not be able to lay up means for return, at least, for some months to come; meanwhile, they might form associations and attachments which would take away all desire to return.

It is a positive waste of money and of means to give in charity on the street, or at your own door. As for feeding people at one's house on certain days of the week, it is a mockery and a pharisaical sham. No; send every ounce of cold food, and every spare rag of cast-off clothing, to the Five Points Missions, or to Mr. Brace, in Astor Place, and then you have the assurance that not an atom will be misappropriated; and as for your money, take every penny you can spare to help your brother man in his need, and purchase tickets from the Association for the Poor, at 39 Bible House; carry these tickets in your pocket, besides having a good supply at your own house, and when application is made for charity, give one of these tickets, which will direct the bearer to a certain place; and on the presentation of that ticket, a responsible person will go with the bearer to his or her home; and if, on minute inquiry, and conversation with neighbors, the person merits aid, it will be given, not in money, but in food, or clothing, or fuel, or rent. These tickets can be had at:

| | |
|---|---|
| 1st Station, 1st & 2d Wards | 27 Greenwich Street. |
| 2d Station, 3th & 5th Wards | 147 Duane Street. |
| 3d Station, 4th & 6th Wards | 106 Centre Street. |
| 4th Station, 7th Ward | 292 Madison Street. |
| 5th Station, 8th & 15th Wards | 129 Thompson Street. |
| 6th Station, 9th Ward | 519 Hudson Street. |
| 7th Station, 10th Ward | 280 Houston Street. |
| 8th Station, 11th Ward | 655 Fourth Street. |
| 9th Station, 13th Ward | 261 Delancy Street. |
| 10th Station, 14th Ward | 142 Grand Street. |
| 11th Station, 16th Ward | 159 West 19th Street. |
| 12th Station, 17th Ward | 442 Fourth Street. |
| 13th Station, 18th Ward | 168 West 23d Street. |
| 14th Station, 20th Ward | 231 West 31st Street. |
| 15th Station, 21st Ward | 418 Third Avenue. |
| 16th Station, 22d Ward | 155 West 43d Street. |

Mr. HARTLEY is the Corresponding Secretary of the "*Association for Improving the Condition of the Poor*," at No. 39 Bible House.

None so well know as physicians, that the real cause of disease and premature death to multitudes in large cities, in winter time, is not the want of money (for, if furnished, it would be improvidently expended, in nine cases out of ten), but it is the want of food and warmth.

These suggestions merit the mature consideration of all who truly sympathize with the poor among us.

